# Rare Complications of Acute Appendicitis: A Case Report

**DOI:** 10.5811/cpcem.2020.11.49601

**Published:** 2021-01-16

**Authors:** Nicholas Kurtzman, Jamie Adler, Andrew Ketterer, Jason Lewis

**Affiliations:** Beth Israel Deaconess Medical Center, Department of Emergency Medicine, Boston, Massachusetts

**Keywords:** Complicated appendicitis, liver abscess, septic thrombophlebitis

## Abstract

**Introduction:**

Appendicitis is a common disease, and as we have improved in early diagnosis and management of this disease process, late stage complications have become extremely rare, but can have indolent presentations.

**Case Report:**

A 37-year-old male with no past medical history presented to the emergency department (ED) with vague abdominal pain as well as 12 days of cyclical fever. He had no significant findings on laboratory workup with the exception of a mild aspartate transaminase and alanine transaminase and relative neutrophilia between outpatient, urgent care, and ultimate ED visit. His ED workup included cross-sectional imaging of his abdomen revealing multiple liver abscesses and septic thrombophlebitis secondary to ruptured appendicitis.

**Conclusion:**

Liver abscesses and septic thrombophlebitis are an extremely rare complication of appendicitis that has only been documented twice previously.

## INTRODUCTION

Appendicitis is a common disease with a lifetime incidence of 8.6% of men and 6.4% of women.^1^ While the disease process typically presents with nausea, anorexia and right lower quadrant tenderness, if this initial phase is missed a host of complications may result, including perforation, abscess formation, and peritonitis. We present a case of a patient with multiple liver abscesses as well as septic thrombophlebitis secondary to ruptured appendicitis. This case highlights a rare phenomenon that has not been documented since the mid-1900s. The patient responded well to antibiotics, interventional radiology (IR)-guided drainage, and interval appendectomy.

## CASE REPORT

A 37-year-old male presented to the emergency department (ED) complaining of 12 days of fever with vague abdominal pain in an “L” shape from the right upper quadrant to the bilateral lower quadrants for which he had already presented to his primary care provider (PCP) and urgent care (UC). Additionally, the patient had a headache at the onset of the illness. He worked as a personal trainer and had not been able to function at his job for about one week due to profound fatigue. His fever was cyclical, occurring approximately every 12 hours and ranging from 101–103.4 degrees Fahrenheit. The patient was initially able to control his fevers with acetaminophen and ibuprofen, but by the time of his ED presentation these therapies failed to achieve effect. He also complained of myalgias. The patient had already had a workup from the PCP and the UC, which were unremarkable with the exception of elevated liver function tests.

At the PCP’s office after three days of fever he had no leukocytosis, no guarding on abdominal exam or abdominal tenderness, and negative testing for Lyme disease, anaplasma, babesia, ehrlichiosis, and influenza. By the time of his UC visit, the patient had been having six days of fevers with a resolution of his initial headache. He had no rash and no guarding on exam, but had diffuse arthralgias. Lab testing revealed no leukocytosis, and a blood smear for parasites was sent that would eventually result as negative. The patient did have an elevation of his alanine aminotransferase and alkaline phosphatase (ALP).

When he presented to the ED, his initial vital signs showed a blood pressure of 149/80 millimeters mercury, a heart rate of 107 beats per minute, a temperature of 99.2ºF, a respiratory rate of 16, and a pulse oximetry reading of 100% on room air. Upon history, the patient had cyclical fevers for the prior 12 days as well as generalized weakness and worsening abdominal discomfort in the right upper quadrant and bilateral lower quadrants. On physical exam, he had intact strength, sensation, and cranial nerve exams, no signs of meningismus, and a non-tender abdomen.

Laboratory values showed a drop in his hemoglobin from 13.3 to 11.0 milligrams per deciliter (mg/dL) (reference range 11.2–15.7 mg/dL), a mild leukocytosis of 12,000 K/uL (4.0–10.0 K/uL) with 84% neutrophils (34–71%) and no bands (0–0.6%), a normal chemistry panel, a lactate of 0.9 millimoles per liter (mmol/L) (0.5–2 mmol/L), an elevated aspartate aminotransferase (AST) of 66 international units per liter (IU/L) (0–40 IU/L), an elevated ALP of 275 IU/L (40–130 IU/L), and a urinalysis showing no evidence of urinary tract infection.

**Image 1:**
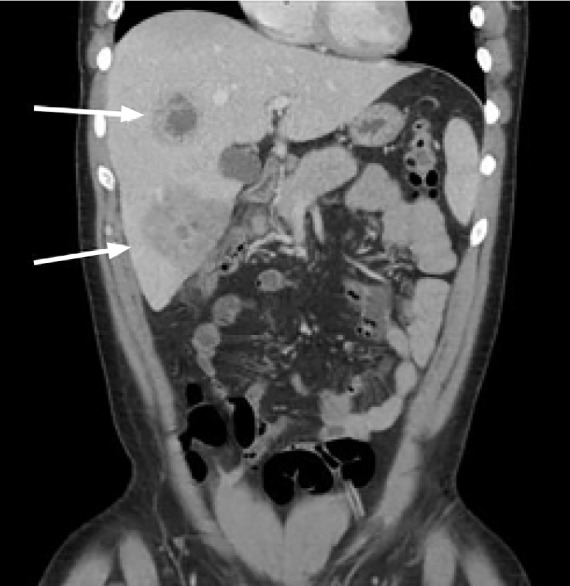
Coronal computed tomography of the abdomen and pelvis of 37-year-old male with intermittent fever and vague abdominal pain with hepatic abscess (arrows).

**Image 2:**
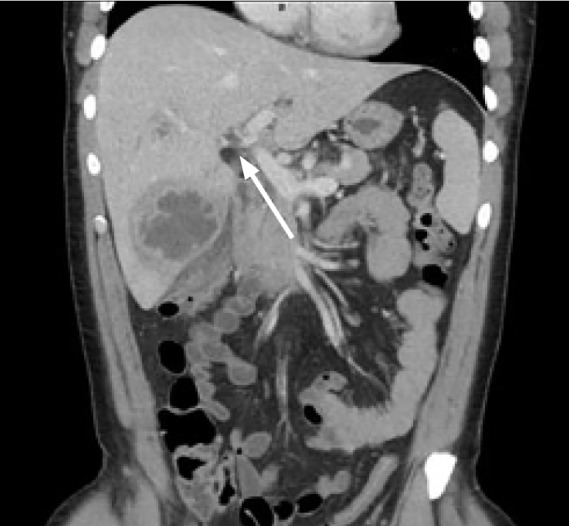
Coronal computed tomography of the abdomen and pelvis of 37-year-old male with intermittent fever and vague abdominal pain with septic thrombophlebitis (arrow).

**Image 3:**
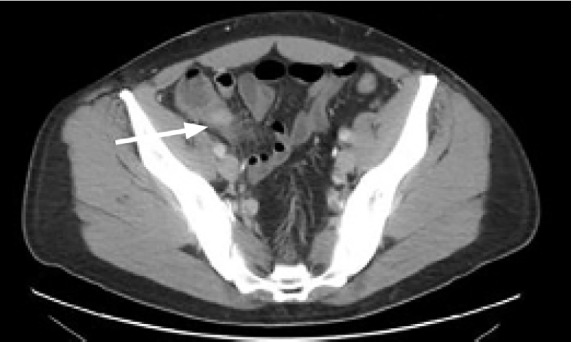
Transverse computed tomography of the abdomen and pelvis of 37-year-old male with intermittent fever and vague abdominal pain with ruptured appendicitis (arrow).

A chest radiograph was negative for signs of pneumonia, after which a computed tomography (CT) of the abdomen and pelvis with intravenous (IV) contrast was obtained, which showed multiple liver abscesses (Image 1), septic thrombophlebitis of the portal system in hepatic segments six and eight (Image 2), and evidence of ruptured appendicitis (Image 3). The patient was initially started on broad spectrum IV antibiotics and within the first 48 hours had IR-guided abscess drainage. Abscess cultures grew out as *Staphylococcus haemolyticus*, which was penicillin sensitive. The patient ultimately underwent an interval appendectomy.

**CPC-EM Capsule**

What do we already know about this clinical entity?

Appendicitis is common, and frequently identified based on a clear constellation of symptoms and exam findings. It is rare that sequelae of perforated appendicitis are encountered.

What makes this presentation of disease reportable?

Appendicitis is not always associated with the classic presentation of right lower quadrant pain, and complications of perforated appendicitis may in fact be the presenting complaint.

What is the major learning point?

When patients present with persistent symptoms and no findings by prior workups, it is important to consider atypical presentations of the common pathologies encountered in the emergency department.

How might this improve emergency medicine practice?

Highlighting uncommon or atypical presentations is a reminder to be cognizant of anchoring bias.

## DISCUSSION

Appendicitis is an extremely common condition affecting 8.6% of males and 6.7% of females, most commonly between ages 10–18 years.^1^ More than 250,000 appendectomies are performed per year in the United States.^2^ Despite the high prevalence of this condition, the complications suffered by our patient are extremely uncommon. As of the 1950s, cases of septic thrombophlebitis of the portal system occurred in approximately 0.4% of cases of appendicitis.^3^ Cases combining liver abscesses and septic thrombophlebitis are even rarer, with only case reports dating back to the 1940s and one other case documented in 2001.^3^–^5^ The 2001 case took six weeks to obtain the definitive diagnosis, which led to further complications of ascites, extensive thrombus formation, and spontaneous bacterial peritonitis secondary to *Escherichia coli* and viridans *Streptococci*. Septic phlebitis and hepatic abscesses can have nonspecific clinical findings such as fever, mild peritoneal findings, nausea, vomiting, or abdominal distention, and often come with normal liver function tests and blood cultures.^5^ We obtained a CT of the abdomen and pelvis due to the patient’s ongoing fever of unknown origin and persistent complaints of abdominal pain and anorexia, despite his benign abdominal exam. The initial expectation was that the patient might have had a malignancy such as lymphoma, given the B symptoms (ie, fevers, night sweats, and weight loss) and lack of a tender abdomen.^6^

While limiting unnecessary CT imaging in younger adults is important,^7^ this case shows that CT imaging can have utility in abdominal pain in a non-tender abdomen with persistent fevers and an otherwise unrevealing workup. Even classic right lower quadrant tenderness is only shown to be sensitive between 50–94% of the time.^8^–^10^ If appropriate imaging is not performed, cases of appendicitis can progress to develop complications involving multiple organs as well as septic thrombi. Once the diagnosis of complicated appendicitis is made on cross-sectional imaging, broad spectrum IV antibiotics should be started, and IR and surgery should be consulted for further management and admission.

## CONCLUSION

This case report presents a rare complication of a common disease process that can present in a multitude of ways. The patient in question had a broad differential that had been worked up previously as an outpatient with non-specific laboratory findings. His only lab abnormalities were AST and ALP elevation and higher-than-normal percentage of neutrophils. Upon presentation to the ED, his vital signs including tachycardia and persistent fever of unknown origin led the clinical team to believe there was underlying pathology that needed additional workup despite his overall well appearance. While the combination of liver abscesses and septic thrombophlebitis is a rare complication of appendicitis, this case demonstrates how appendicitis can mimic other disease states with non-specific signs and symptoms, and diagnosis can be consequently delayed leading to downstream complications.
